# IssuEs in Palliative care for people in advanced and terminal stages of Young-onset and Late-Onset dementia in GErmany (EPYLOGE): the study protocol

**DOI:** 10.1186/s12888-018-1846-0

**Published:** 2018-08-31

**Authors:** Janine Diehl-Schmid, Julia Hartmann, Carola Roßmeier, Lina Riedl, Hans Förstl, Silvia Egert-Schwender, Victoria Kehl, Helga Schneider-Schelte, Ralf J. Jox

**Affiliations:** 1 Department of Psychiatry and Psychotherapy, Technical University of Munich, Ismaninger Str.22, 81675 Munich, Germany; 2 Münchner Studienzentrum, Technical University of Munich, Munich, Germany; 3 Institute for Medical Informatics, Statistics and Epidemiology of Technical University of Munich, Munich, Germany; 4 German Alzheimer Society, Berlin, Germany; 5 Institute of Ethics, History, and Theory of Medicine, Ludwig Maximilians Universität, Munich, Germany; 6 Geriatric Palliative Care, Department of Medicine, Lausanne University Hospital, Lausanne, Switzerland

**Keywords:** Dementia, Young onset dementia, Early onset dementia, Palliative care, Quality of life, End-of-life, Dying

## Abstract

**Background:**

Scientific research on palliative care in dementia is still underdeveloped. In particular, there are no research studies at all on palliative care issues in young onset dementia (YOD), although significant differences compared to late onset dementia (LOD) are expected. Most studies have focused on persons with dementia in long term care (LTC) facilities but have neglected persons that are cared for at home. We hypothesize that unmet care needs exist in advanced and terminal stages of YOD and LOD and that they differ between YOD and LOD.

**Methods/design:**

The EPYLOGE-study (IssuEs in Palliative care for people in advanced and terminal stages of Young-onset and Late-Onset dementia in GErmany) aims to prospectively assess and survey 200 persons with YOD and LOD in advanced stages who are cared for in LTC facilities and at home. Furthermore, EPYLOGE aims to investigate the circumstances of death of 100 persons with YOD and LOD. This includes 1) describing symptoms and management, health care utilization, palliative care provision, quality of life and death, elements of advance care planning, family caregivers’ needs and satisfaction; 2) comparing YOD and LOD regarding these factors; 3) developing expert-consensus recommendations derived from the study results for the improvement and implementation of strategies and interventions for palliative care provision; 4) and communicating the recommendations nationally and internationally in order to improve and adapt guidelines, to change current practice and to give a basis and perspectives for future research projects. The results will also be communicated to patients and their families in order to counsel and support them in their decision making processes and their dialogue with professional caregivers and physicians.

**Discussion:**

EPYLOGE is the first study in Germany that assesses palliative care and end-of-life issues in dementia. Furthermore, it is the first study internationally that focuses on the specific palliative care situation of persons with YOD and their families. EPYLOGE serves as a basis for the improvement of palliative care in dementia.

**Trial registration:**

The study is registered in ClinicalTrials.gov (NCT03364179; Registered: 6. December 2017.

## Background

Most dementias are life-limiting disorders for which a curative treatment does not exist. Advanced stages of dementia are characterized by severe cognitive and physical impairment and the need for 24-h care for most patients. Therefore, at least 50% of persons with advanced dementia are admitted into long term care (LTC) settings, where they finally die if they are not transferred to an acute hospital shortly before their death [1, 2].

It is controversial when palliative care should start during the disease trajectory. However, it is beyond doubt that in advanced dementia stages - when the aim of treatment is no longer focused on stabilization of cognitive functioning nor prolongation of life - provision of comfort-oriented measures in order to maximize quality of life requires palliative care, sometimes even specialized palliative care [3]. Worldwide – and particularly in the Netherlands and Great Britain - several research groups have been focusing on palliative care in dementia. The intersection of palliative care and late-onset dementia (LOD) has been addressed in numerous studies, e.g. the *Dutch End of Life in Dementia (DEOLD*) study [4], the *Implementation of quality indicators in palliative care study (IMPACT)* [5] and the *Zürich Life and Death with Advanced Dementia* (ZULIDAD) study [6]. These studies provide insights into the symptoms and findings observed in advanced and terminal stages of dementia, treatment options, healthcare conditions, quality of life and death, patients’ needs, care goals, reasons and circumstances of death, end-of-life outcomes, attitudes of nursing staff and doctors as well as the needs and problems of relatives, formal and informal caregivers. Various instruments have been validated in order to assess pain, burden, discomfort, and quality of life (e.g. [7]) and have successfully been used as outcome measures [8–13].

In several countries, guidelines or recommendations have been developed in order to improve the delivery of palliative care for persons with dementia. As an example, the guidelines on dementia of the *British National Institute on Clinical Excellence* [14] include key palliative care components. The *European Association for Palliative Care (EAPC)* formulated specific recommendations for palliative care of persons with dementia [3].

Compared to the multifaceted international efforts to evaluate and improve palliative care strategies in dementia, research initiatives and care strategies in Germany are scarce. Initiated by the *Robert-Bosch-Stiftung*, a *Curriculum Palliative Practice* for staff that works in old-age care with persons suffering from dementia was developed. The *German Society for Palliative Care* has established a standardized training of teachers for the “*Curriculum Palliative Practice”,* though effects of the training have not yet been evaluated.

A few reviews on palliative care and dementia have been conducted by German authors [15]. One study has investigated causes and places of death of persons with dementia in Germany [2]. The authors describe that persons with advanced dementia suffer from several symptoms that may need specialized palliative care, whether it be at home, within a nursing home or in a hospital.

Given the significant lack of research in the field of palliative care and dementia in Germany, it is impossible to give evidence-based recommendations or to even develop and evaluate interventions in order to improve palliative care. Palliative care depends not only on cultural, ethical and social prerequisites but also on legal and financial conditions in different countries. Hence, it is almost impossible to transfer research results, such as those from the numerous, high-quality international studies, to persons with dementia in Germany.

Neither in Germany nor internationally has any research been conducted on palliative care in young onset dementia (YOD), defined by an age at symptom onset before 65 years, who live at home or in long term care (LTC) facilities. Significant differences are expected between YOD compared to late-onset dementia (LOD), such as lower somatic comorbidities in YOD, less frailty, more behavioral and psychological symptoms, different responses to psychopharmacological therapy, different attitudes regarding end-of-life, and higher caregiver burden [16]. However, these hypotheses have yet to be confirmed. Furthermore, most international studies have focused on palliative care provision in LTC settings, leaving little knowledge about palliative care of persons with advanced dementia who receive care at home.

### Aim and research questions

Therefore, the aims of the prospective cohort study EPYLOGE are:

1. To prospectively assess and describe advanced and terminal stages of dementia patients who live in LTC facilities or at home, regarding the following criteria: a) symptom burden and symptom management, b) health care utilization, c) palliative care provision, d) quality of life and quality of death, e) family caregivers’ satisfaction and needs regarding care and decision making; f) advance care planning; g) validity and applicability of existing advance directives for the situation of dementia.
2. To compare YOD and LOD: we hypothesize that in advanced stages and at the end of life of YOD and LOD patients, problems and unmet care needs exist and differ between YOD and LOD.
3. To develop expert-consensus recommendations for the improvement and implementation of strategies/ interventions regarding palliative care provision for persons with advanced YOD and LOD.
4. To communicate the results and the consecutive recommendations both nationally and internationally to the scientific community, to relevant parties that are involved in patient care and to patients and their families.

## Methods

EPYLOGE is a prospective cohort study that includes a baseline visit with the person suffering from dementia and a family caregiver, quarterly phone calls with the family caregivers and a follow-up visit with the family caregiver after the person with dementia has died.

### Subjects

Dyads of persons with advanced dementia, who live at home or in LTC, and their family caregivers are identified in multiple ways: 1) Through the Center of Cognitive Disorders at the University Hospital of the Technical University of Munich: The family caregivers of persons with a diagnosis of a neurodegenerative dementia, who have been seen in the outpatient clinic since 2005, are contacted. If the person with dementia fulfills the inclusion criteria, he or she, and the corresponding family caregiver will be invited to participate in the study; 2) Through a collaborative network specialized in YOD in order to include a sufficiently high number of persons with YOD (in spite of its relatively low prevalence), institutions specialized in YOD (i.e. nursing homes, support centers, research centers, etc.) are asked to establish contact with families of persons with advanced YOD; 3) through LTC units in urban and rural areas of Germany. Lack of informed consent either from the person with dementia (or their legal surrogate) or from their family caregiver is an exclusion criterion.

### Study design

**Fig. 1:**
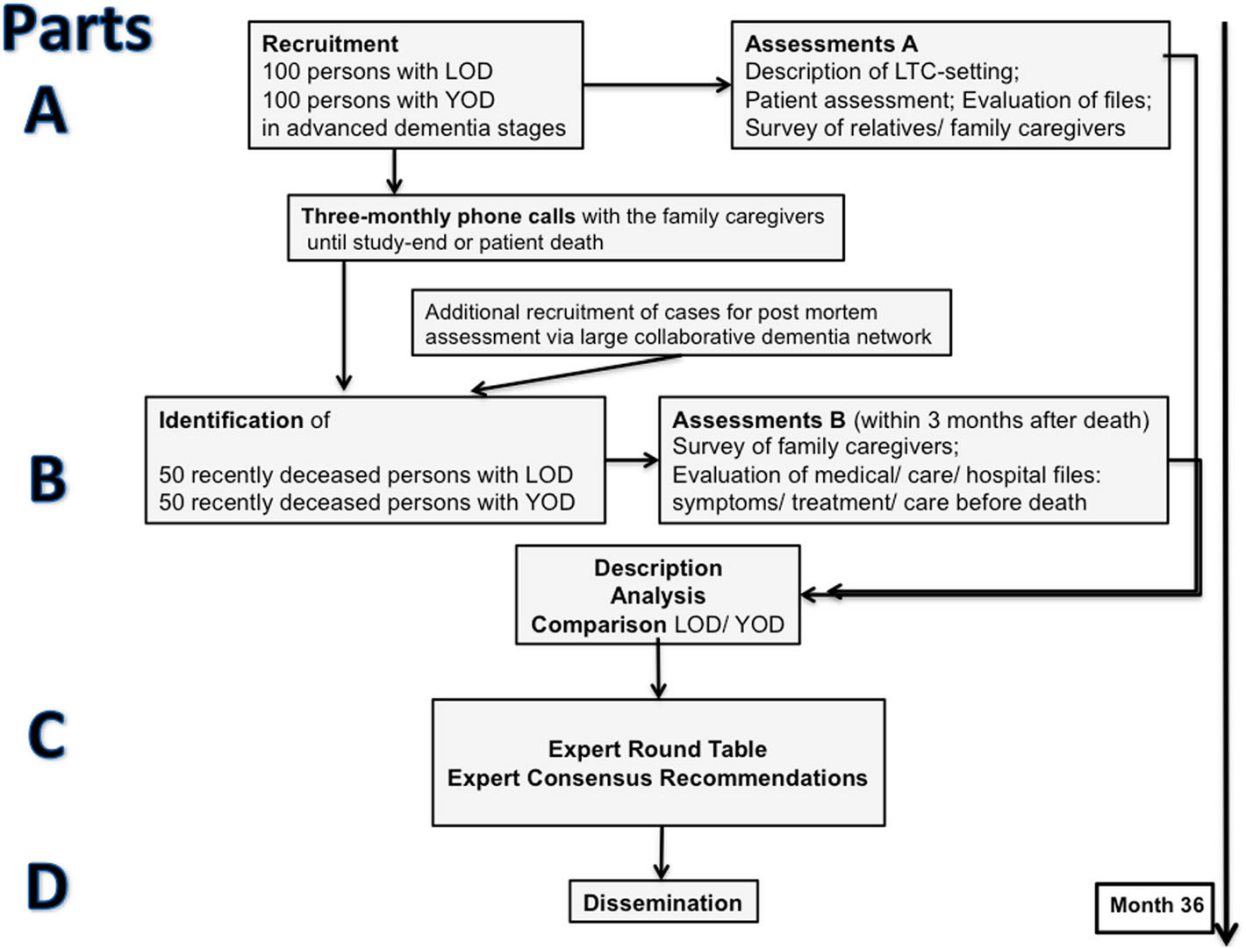
EPYLOGE: Flow chart of study design and procedures

EPYLOGE consists of four parts (see Fig. 1):

In **part A** (baseline-visit), palliative care issues in advanced dementia are analyzed and compared between 100 persons with YOD and 100 persons with LOD. Studies have shown that in Germany about 50% of persons with advanced dementia are cared for at home [2]. Thus, the objective is to include approximately 50 persons living at home and 50 persons living in LTC in each group.

After the baseline visit, family caregivers are contacted by phone every three months in order to briefly assess changes of the living and care situation. The phone calls are continued until the death of the person with dementia or until the end of study. After the patient’s death assessment B will be performed within 3 months at the latest.

In **part B** (follow-up visit) palliative care issues at the end of life are analyzed and compared between 50 deceased persons with YOD and 50 deceased persons with LOD identified by the phone calls described above. It is anticipated that about 50% of the participants in part A will die during the study period. In case this assumption is not true and fewer participants die, or if deaths are more common in specific subgroups (age, living situation), the groups in part B are completed with additional bereaved families.

In **part C**, the results of part A and B will be discussed in an expert round table that is scheduled for early 2020. Expert consensus recommendations will concern strategies and interventions in order to improve palliative care in YOD and LOD.

In **part D** the expert consensus recommendations will be disseminated.

### Data collection

The first dyad comprised of the person with advanced dementia and the family caregiver was included in October 2017. Patient recruitment is expected to last until December 2019.

### Instruments and measures

For **part A** - palliative care issues in advanced dementia: a detailed assessment of the person with advanced dementia and a standardized interview of the family caregiver will be conducted at the baseline visit. If necessary, additional information will be gathered from the nursing staff. The instruments and measures of part A are described in Table 1.

**Table 1:** Part A, baseline visit: instruments and measures

Assessments of the person with dementia	
General data: Demographic data, living and care situation	
Etiology of dementia, date of symptom onset, date of dementia diagnosis	
Psychiatric and somatic comorbidities	
Medical care: Pharmacotherapy, tube feeding, custodial measures	
Cognitive status: Mini-Mental Status Examination (MMSE); Brief Language Assessment	
Severity of dementia: Clinical Dementia Rating (CDR)	
Activities of daily living: Barthel-Index	
Pain: Pain Assessment in Advanced Dementia (PAINAD) Scale	
Comfort: Discomfort Scale in Dementia (DS-DAT)	
Patient suffering: Mini Suffering State Examination (MSSE)	
Patient examination: Neurologic and somatic symptoms; psychopathology	
Assesment of palliative care provision	
Assessment of advance care planing (palliative care goals, advance directives, emergency orders)	
Additional information about the person with dementia obtained from the family caregiver and, if necessary, from the nursing staff (if applicable)	
Quality of life: Quality of Life in Late Stage Dementia (QUALID)	
Symptom management: Modified version of End of Life in Dementia-Symptom Management/ EOLD-SM	
Behavioral and psychological symptoms in dementia: Neuropsychiatric Inventory (NPI)	
Suicidal ideation and behavior	
Interviews of the family caregiver	
General data: Demographic data, living situation	
Wellbeing: WHO Five (WHO-5)	
Depression: Beck Depression Inventory II (BDI II)	
Strain: Caregiver Strain Index (CSI) (Robinson et al., 1983)	
Burden: Burden Scale for Family Caregivers (BSFC)	
Perception of care: Family Perception of Care Scale of St. Michael’s Hospice North Hampshire (FPCS) (https://www.stmichaelshospice.org.uk/NHSS_family_perceptions_of_care_scale.pdf)	
Satisfaction with care: Modified version of End of Life in Dementia-Satisfaction with Care (EOLD-SWC)	
Open questions: Caregivers’ problems, challenges, barriers, needs, preferences regarding palliative care	
Description of the LTC facility	
If the person with dementia lives in an LTC facility, the administrator is asked to provide a detailed and standardized description of the facility, infrastructure, organization, service provision, and palliative care provision.	

After part A, the respective family caregiver is perdiocially phoned every three months and changes regarding living situation, care provision and comorbidities are assessed. The post-mortem visit (part B) is arranged if the person with dementia has died.

In **part B** - end-of life issues in advanced dementia: standardized post mortem assessments are performed with the family caregivers of 50 persons with YOD and 50 persons with LOD at a follow-up visit.

In part B, a standardized interview with the bereaved family caregiver is performed less than three months after the person with dementia has died. Instruments and measures are described in Table 2.

**Table 2:** Part B, follow-up visit with family caregiver post-mortem: instruments and measures

Interviews of the family caregiver	
Symptoms, treatment and care before death and circumstances of death as reported by the caregiver and identified by retrospective evaluation of medical and care files; evaluation if advance directives have been applied.	
Caregiver satisfaction with care: End of Life in Dementia-Satisfaction with Care (EOLD-SWC) (Volicer L et al., [8])	
Burden: Modified version of Burden Scale for Family Caregivers (BSFC)	
Patient suffering: Mini Suffering State Examination (MSSE)	
Quality of life: Modified version of Quality of Life in Late Stage Dementia (QUALID)	
Symptom management: End of Life in Dementia-Symptom Management (EOLD-SM) (Volicer L et al., [8])	
Persons with dementias comfort in dying: Comfort Assessment of Dying with Dementia (CAD-EOLD) (Volicer L et al., [8])	
Caregiver wellbeing: WHO five (WHO-5)	
Caregiver depression: Beck Depression Inventory II (BDI II)	
Open questions: Caregivers’ problems, challenges, barriers, needs, preferences regarding end-of-life care.	
Description - LTC	
If the person with dementia lives in an LTC facility, the administrator is asked to provide a detailed and standardized description of the facility, infrastructure, organization, service provision, and palliative care provision.	

**Part C:** After parts A and B are completed, an **expert round table discussion** will be organized with experts from the fields of dementia, palliative medicine and LTC care, patient advocacy groups, dementia and palliative care specialists, a medical ethicist, a jurist with a focus on end-of-life topics, general practitioners providing general outpatient palliative care representatives from specialized outpatient palliative care services, from the Health ministries as well as representatives from health/nursing insurance companies. The results of parts A and B will be reported and discussed in comparison to the findings of international studies concerning the same topic. Expert consensus recommendations will be drafted for strategies and interventions in order to improve palliative care in YOD and LOD.

**Part D: Dissemination**: The results of the study and of the expert consensus recommendations will be communicated nationally and internationally to the scientific community in order to improve and adapt care guidelines as well as to give a basis and perspective for future research projects. Furthermore, the results and recommendations will be communicated to the relevant parties that are involved in patient care and support (family and professional caregivers, palliative care specialists, physicians, memory clinics, patient advocacies, LTC administrators, Ministries of Health, and health/nursing insurance) in order to implement the recommendations into daily care practice. Thirdly, the results and recommendations will be communicated to persons with dementia and their families (through lay press, lay people events, radio, internet, brochure) in order to counsel and support their decision-making processes and their communication with professional caregivers and physicians.

### Endpoints and sample size calculation

The scales and questionnaires in this study have been used in several international studies investigating palliative care in dementia, including the Netherland’s Dutch End of Life (DEOLD) study. A recent review has indicated good psychometric properties in tests of palliative care such as the *Discomfort Scale for Dementia of the Alzheimer Type (DS-DAT),* which offers high internal consistency [17]*.*

Primary outcomes of EPYLOGE include patients’ quality of life, as measured by the *Quality of Life in Late Stage Dementia-Scale (QUALID)* [18], and quality of death, as measured by the *Comfort Assessment of Dying with Dementia-Scale (CAD-EOLD)* [8]). These assessments of quality of life and death will be compared between YOD and LOD patients. Secondary outcomes include symptom burden and symptom management, comfort measures, caregivers’ burden, satisfaction with care and shared-decision making as well as health care utilization. These secondary outcomes will be measured by a variety of scales and questionnaires. The impact of living situation (home/nursing home), gender, social status and residence (rural vs. metropolitan area) on the primary endpoints will be assessed.

Sample size calculation for the comparison of persons with YOD and LOD was performed using the primary endpoints. Bonferroni adjustment for multiple comparisons was utilized. The global significance level was set to 5%. For part A, the comparison of persons with YOD and LOD in advanced stages of dementia, the quality of life, as measured by the *Quality of Life in Late Stage Dementia-Scale (QUALID)* [18]), was chosen as the primary endpoint. Scale characteristics include: a near-normal distribution, a range of 11 to 55 in which lower scores reflect higher quality of life during the last seven days of life, a mean of 22.76, a standard deviation of 7.40, and a hypothesized clinically meaningful difference of 5 points. A sample size of 86 persons with dementia within each group will have 89% power in detecting a difference in mean QUALID scores of 4, assuming that the common standard deviation is 7.4 and that the two-sided independent samples t-test used will have 2.5% local significance. Assuming a drop-out rate of 14%, at least 100 persons per group will be required, comprising a total of 200 persons.

For part B, the comparison of persons with YOD and LOD at end of life, the quality of death, as measured by the *Comfort Assessment of Dying with Dementia-Scale (CAD-EOLD)* [8]), was chosen as the primary endpoint. Scale characteristics include: a near-normal distribution, a range of 14 to 42 in which higher scores reflect better comfort level, a mean of 31.4, a standard deviation of 5.9, and a hypothesized clinically meaningful difference of 3 points. A sample size of 43 persons with dementia in each group will have 80% power in detecting a difference in mean CAD-EOLD scores of 4, assuming that the common standard deviation is 5.9 and that the two-sided independent samples t-test used will include 2.5% local significance. Assuming a drop-out rate of 14%, at least 50 recent cases of deceased persons per group will be required for part B.

### Statistical analysis

Primary endpoint analysis: The two groups (YOD and LOD) will be compared with respect to both primary endpoints. The two-sided independent samples t-test will be used to test for differences in both primary endpoints. The global significance level will be set to 5%. The local significance level of each test (Bonferroni adjustment) will therefore be 2.5%.

Secondary endpoint analysis: Analyses of baseline data and secondary endpoints will be performed using appropriate descriptive statistics and independent samples tests for differences between the two study groups. All tests will be two-sided with an exploratory significance level of 5%.

### Ethical considerations, quality assurance and data protection

The study was approved by the Ethics Committee of the Faculty of Medicine of the Technical University of Munich (18. Aug. 2017; No. 281/17 S). A written informed consent procedure with the patients and/or their legal surrogates and the family caregivers is the basis for study participation. The multi-disciplinary International Scientific Advisory Board considers scientific and ethical issues. National and international standards of quality assurance, particularly the Declaration of Helsinki Note for Guidance on Good Clinical Practice (GCP) of the ICH (ICH-GCP), 17.1.1997; (European Medicines Agency, 2002) are respected. Monitoring will be performed according to a study-specific monitoring manual and GCP-compliant monitoring standard operating procedures. The study is conducted in compliance with German data protection guidelines. Data are handled confidentially. All information related to the study will be kept archived for at least 10 years after completion of the study in accordance with §13 Sec. 10 of the GCP Regulations. The study is registered in ClinicalTrials.gov (NCT03364179; registered: 06. Dec 2017).

## Discussion

To our knowledge, EPYLOGE is the first study that addresses palliative care issues concerning dementia in Germany. A unique feature of EPYLOGE is its focus on YOD and the comparison between YOD and LOD. Some limitations of the study need to be considered. Firstly, there may be a sampling bias; most patients will be recruited through the memory clinic of a university hospital. Patients who receive treatment from a tertiary care center may not be representative for the general population. However, we believe that recruitment through LTC facilities - as done in most prior studies - generates an even larger sampling bias. That is because some LTC facilities, presumably those who feel that they are deficient in palliative care, may refuse to participate. Secondly, most of the patient-related data will be collected by interviewing family caregivers and nursing staff. These data may be less reliable than desired; however, asking patients with advanced dementia directly about their condition, complaints, etc. would potentially generate less reliable data and is ethically problematic. Thirdly, the first visit might raise awareness in the caregivers and perhaps in the nursing staff. As a consequence, palliative care may be improved. Therefore, a bias should be considered at the post mortem visit. Patients might have received better care at the end of their life and experienced a better quality of death than they would have if they had not been included in part A of EPYLOGE.

In order to avoid wrong conclusions, one must consider the limitations of the study during the interpretation and dissemination of results. EPYLOGE aims to address these limitations throughout the study, allowing for stronger interpretations of results. In summary, EPYLOGE will serve as a basis for the improvement of palliative care in dementia. EPYLOGE also aims to implement strategies and interventions regarding palliative care provision for persons with advanced YOD and LOD. Furthermore, EPYLOGE is a cornerstone of future research in the field of palliative care in dementia.

## Abbreviations

BDI II: Beck Depression Inventory II
BMBF: Bundesministerium für Bildung und Forschung, German Ministry for Education and Research
BSFC: Burden Scale for Family Caregivers
CAD-EOLD: Comfort Assessment of Dying with Dementia
CDR: Clinical Dementia Rating
CSI: Caregiver Strain Index
DEOLD: Dutch End of Life in Dementia
DS-DAT: Discomfort Scale in Dementia
EAPC: European Association for Palliative Care
EOLD-SM: End of Life in Dementia-Symptom Management
EOLD-SWC: End of Life in Dementia-Satisfaction with Care
EPYLOGE: IssuEs in Palliative care for people in advanced and terminal stages of Young-onset and Late-Onset dementia in Germany
FPCS: Family Perception of Care Scale of St. Michael’s Hospice North Hampshire
IMPACT: Implementation of quality indicators in palliative care study
LOD: Late onset dementia
LTC: Long term care
MMSE: Mini-Mental Status Examination
MSSE: Mini Suffering State Examination
NPI: Neuropsychiatric Inventory
PAINAD: Pain Assessment in Advanced Dementia
QUALID: Quality of Life in Late Stage Dementia
SAPV: Spezialisierte ambulante Palliativversorgung, specialized outpatient palliative care
WHO-5: WHO Five
YOD: Young onset dementia
ZULIDAD: Zürich Life and Death with Advanced Dementia
